# *CsCBDAS2*-Driven Enhancement of Cannabinoid Biosynthetic Genes Using a High-Efficiency Transient Transformation System in *Cannabis sativa* ‘Cheungsam’

**DOI:** 10.3390/plants14101460

**Published:** 2025-05-14

**Authors:** Sang-Cheol Baek, Sang-Yoon Jeon, Bo-Hyun Byun, Da-Hoon Kim, Ga-Ram Yu, Hyuck Kim, Dong-Woo Lim

**Affiliations:** 1 TOPO Lab., Co., Ltd., Goyang 10326, Republic of Korea; 9494bbaek@gmail.com (S.-C.B.); panax1218@gmail.com (S.-Y.J.); macbo2001@gmail.com (B.-H.B.); lilwayne224@gmail.com (D.-H.K.); kalama2@dongguk.edu (G.-R.Y.); hyuckkim@dongguk.ac.kr (H.K.); 2 Department of Life Science, Dongguk University, Goyang 10326, Republic of Korea; 3 Department of Diagnostics, College of Korean Medicine, Dongguk University, Goyang 10326, Republic of Korea; 4 Institute of Korean Medicine, Dongguk University, Goyang 10326, Republic of Korea

**Keywords:** transient transformation, agroinfiltration, cannabinoid, *Cannabis sativa* Cheungsam, qPCR, GUS

## Abstract

*Cannabis sativa* produces pharmacologically valuable cannabinoids. In this study, we developed and optimized a transient transformation system using *Cannabis sativa* ‘Cheungsam’ to facilitate gene functional analysis. Various experimental conditions, including plant developmental stages, light conditions, *Agrobacterium* strains, tissue types, and physical treatments such as sonication and vacuum infiltration, were systematically evaluated using GUS histochemical staining and qPCR analysis. Among these, 7-day-old seedlings cultured under dark conditions and transformed with the GV3101 strain exhibited high transformation efficiency. Leaf tissue showed a higher GUS staining proportion and GUS staining area compared to hypocotyl and cotyledon tissues. The application of a combination of sonication and vacuum infiltration techniques resulted in the most intense GUS expression. Using the optimized protocol, we introduced a recombinant vector carrying *CsCBDAS2*, a key gene in cannabidiol (CBD) biosynthesis. qPCR analysis revealed that *CsCBDAS2* overexpression led to significant upregulation of multiple upstream CBD biosynthetic genes (*CsOAC*, *CsGOT*, *CsPT1*, *CsPT4*, *CsCBDAS1*, and *CsCBDAS2*) and the transcription factor (TF) *CsWRKY20*, suggesting coordinated co-expression and potential involvement of a transcriptional feedback loop. These results demonstrate the effectiveness of our transient transformation system and provide insights into the regulatory mechanisms of cannabinoid biosynthesis in *cannabis*.

## 1. Introduction

*Cannabis* is an annual, wind-pollinated dioecious plant classified within the Cannabaceae family [1,2,3]. It is presumed to have originated in Asia and has been used as a medicinal and fiber resource since approximately 2700 B.C. [4]. *Cannabis* is primarily classified into three subspecies: *Cannabis sativa*, *Cannabis indica*, and *Cannabis ruderalis*. However, due to the anemophilous nature of *Cannabis*, frequent natural hybridization has led to the identification of more than 600 variants worldwide [5,6].

*Cannabis* contains over 100 cannabinoids, which are unique secondary metabolites comprising an alkylresorcinol and a monoterpene moiety [7]. The primary compounds include ∆^9^-tetrahydrocannabinol (THC) and cannabidiol (CBD). THC is the principal psychoactive component of *Cannabis*, exerting its effects on the central nervous system to induce mood alterations, pain relief, appetite stimulation, and muscle relaxation [8]. Conversely, CBD is the non-psychoactive compound with antioxidative and anti-inflammatory properties, demonstrating neuroprotective effects in both acute and chronic neurodegenerative diseases. Additionally, CBD has been utilized in the treatment of epilepsy, anxiety disorders, and pain management [9,10].

**Figure 1:**
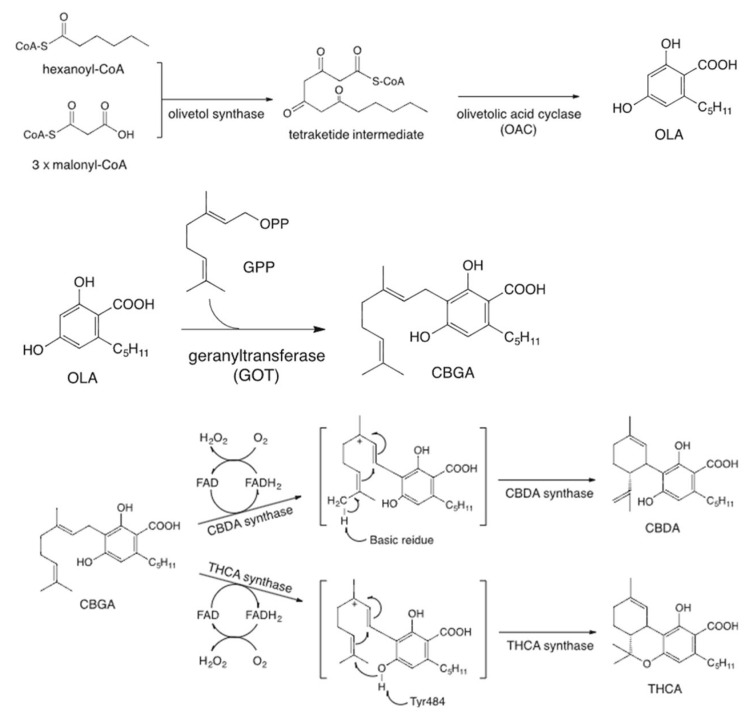
Cannabinoid biosynthesis pathway.

Since the mid-1990s, researchers have extensively investigated the enzymes involved in cannabinoid biosynthesis. Plant-derived cannabinoids are classified into two types: neutral cannabinoids and cannabinoid acids, depending on the presence of a carboxyl group. In *Cannabis* plants, cannabinoids are primarily biosynthesized and accumulated in the form of cannabinoid acids, which are non-enzymatically decarboxylated into their neutral forms during storage or heating [11,12]. Both cannabidiolic acid (CBDA) and tetrahydrocannabinolic acid (THCA) are enzymatically derived from a common precursor, cannabigerolic acid (CBGA), through the action of specific oxidoreductases, CBDA synthase (CBDAS) and THCA synthase (THCAS), respectively. CBGA is synthesized via an alkylation reaction between olivetolic acid (OA) and geranyl pyrophosphate (GPP), a process catalyzed by a prenyltransferase enzyme (Figure 1) [13].

CBDAS is a flavin adenine dinucleotide (FAD)-dependent oxidase that catalyzes the oxidative cyclization of CBGA in the presence of molecular oxygen to yield CBDA [14]. CBDAS shares approximately 84% amino acid sequence identity with THCAS, and both enzymes are believed to have originated from a common ancestral gene [15]. While they exhibit similar biochemical properties and catalytic mechanisms, their product specificity is determined by subtle differences in the active site residues. Recent studies have revealed the presence of multiple isoforms and paralogs within the CBDAS gene family, suggesting that this family has evolved through gene duplication and diversification [15,16]. A recent study has demonstrated that the activation of cannabinoid biosynthesis in *Cannabis sativa* trichomes is intricately regulated by transcription factors, including WRKY, HD-ZIP, MYB, and bHLH, highlighting their pivotal role in modulating gene expression networks responsible for secondary metabolite production [17].

Transient transformation is a widely utilized technique in plant biotechnology that enables the rapid and temporary expression of foreign genes in plant tissues without stable genomic integration [18]. This approach is particularly advantageous for functional gene analysis, protein production, and metabolic engineering, as it circumvents the time-consuming process of generating stable transgenic lines [18,19]. *Agrobacterium*-mediated transient transformation relies on *Agrobacterium* to deliver the transfer DNA (T-DNA) region of its Ti plasmid into plant cells, leading to high expression levels of target genes within a short period [20]. This method has been successfully applied in numerous plant species for large-scale protein production, virus-induced gene silencing (VIGS), and functional genomics studies [21].

Recent advancements in transient transformation technologies have expanded their applications across various fields, including synthetic biology, plant-based protein production, and genome editing. In particular, virus vector-based transient expression systems utilizing replication elements derived from Tobacco mosaic virus (TMV) and geminiviruses have significantly enhanced recombinant protein expression levels in plants, enabling large-scale production [22,23]. Moreover, the introduction of the CRISPR/Cas9 system through transient transformation has facilitated highly efficient genome modifications, making it a crucial strategy in functional genomics research and crop improvement [24].

*Cannabis sativa* ‘Cheungsam’ is a cultivar developed through the hybridization of the landrace *Cannabis sativa*, the low-THC IH3 variety introduced from the Netherlands in 1997, and a domestic Korean variety [25]. The cultivar was officially registered with the Korea Seed & Variety Service in 2002 (registration number: 05-0015-2002-01). Widely used in Korea for various industrial purposes, *Cannabis sativa* ‘Cheungsam’ is characterized by its low narcotic contents, with THC and CBD levels of 0.34% and 1.34%, respectively [6].

Due to its high flexibility and efficiency, transient transformation has become an indispensable tool in plant molecular biology and biotechnology. However, to date, no systematic study has been conducted on *Agrobacterium*-mediated transient transformation using the infiltration method in *Cannabis sativa* ‘Cheungsam’.

In this study, we aimed to optimize the transient transformation conditions for *Cannabis sativa* by evaluating various physical and biological parameters to enhance transformation efficiency. Furthermore, using the optimized protocol, we introduced a recombinant vector containing *CsCBDAS2* to investigate its impact on CBD biosynthesis-related gene expressions.

Overall, the findings of this study provide a foundation for transient transformation research in *Cannabis* and are expected to broaden its application in plant biotechnology, particularly for the rapid functional validation of genes involved in cannabinoid biosynthesis.

## 2. Results

### 2.1. Optimized Transient Transformation Protocol for Cannabis sativa ‘Cheungsam’

#### 2.1.1. Developmental Stage

To determine the optimal efficiency of transient transformation, experiments were conducted at different developmental stages of the plant (7-day-old and 21-day-old), followed by histochemical GUS analysis (Figure S1a–d). The results showed that 69.87% of the 7-day-old plants exhibited GUS staining, while 69.26% of the 21-day-old plants showed staining (Figure S1e). In addition, the stained area on the leaf surface was quantified using ImageJ 1.54g software. The analysis revealed that the blue-stained area in the 7-day-old plants was approximately 20% larger than that in the 21-day-old plants (Figure S1f). However, there was no statistically significant difference between the two groups. Therefore, both developmental stages were considered suitable for transient transformation in *Cannabis sativa* ‘Cheungsam’. However, given the nature of transient transformation experiments, which aim for rapid results, 7-day-old plants were used for subsequent experiments.

#### 2.1.2. Light Condition

To enhance the efficiency of transient transformation, plants were cultured under two different light conditions: a 16 h/8 h (light/dark) photoperiod and continuous darkness. Histochemical GUS analysis was then performed to evaluate transformation efficiency (Figure S2a–d). The results showed that 87.5% of the plants cultured under the 16 h/8 h (light/dark) condition exhibited GUS staining, while 83.67% of the plants grown in continuous darkness exhibited staining (Figure S2e). No statistically significant difference was observed between the two groups. In addition, the percentage of blue-stained area on the leaf surface was quantified using ImageJ software. The analysis revealed that plants cultured in continuous darkness exhibited approximately 5% more stained area than those grown under the 16 h/8 h (light/dark) condition (Figure S2f). However, similar to the GUS staining rate, this difference was not statistically significant. Nonetheless, plants grown in continuous darkness exhibited a more intense blue coloration compared to those grown under the 16 h/8 h (light/dark) condition. Therefore, continuous darkness was considered the optimal light condition for *Cannabis sativa* ‘Cheungsam’ transient transformation, and plants grown under dark conditions were used in subsequent experiments.

#### 2.1.3. Agrobacterium Strains

**Figure 2:**
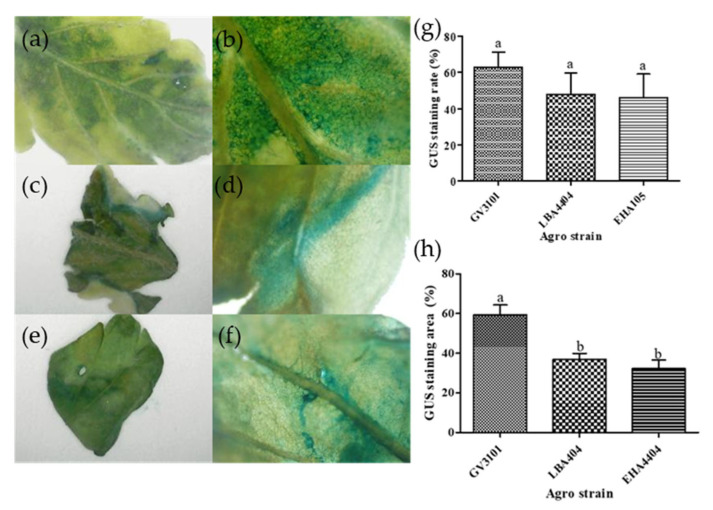
Histochemical GUS staining in *Cannabis sativa* ‘Cheungsam’ tissues transformed with different *Agrobacterium tumefaciens* strains. The blue-stained tissue indicates the presence of GUS activity. (**a**–**f**) Representative images showing GUS expression patterns in transformed tissues. (**a**,**b**) GV3101/pDS-*GUS*; (**c**,**d**) LBA4404/pDS-*GUS*; (**e**,**f**) EHA105/pDS-*GUS*; (**g**) Percentage of GUS-stained seedlings transformed with different *Agrobacterium* strains; (**h**) Quantification of GUS-stained area in tissues using ImageJ (version 1.54g). Data represent the means of three replicates, and error bars indicate SEM. Means with the same letters are not significantly different (Tukey’s test, *p* < 0.05).

A histochemical GUS assay was performed to compare the efficiency of three different *Agrobacterium* strains—GV3101, LBA4404, and EHA105—harboring the pDS-GUS binary vector (Figure 2a–f). Among the tested strains, GV3101 exhibited the highest staining proportion at 62.99%, followed by LBA4404 at 47.96% and EHA105 at 46.00%. Although GV3101 showed approximately 1.3 times higher staining efficiency compared to the other two strains, the difference was not statistically significant (Figure 2g). In addition, the blue-stained area on the leaf surface was quantitatively analyzed using ImageJ software. The results showed that the plants treated with GV3101 had the highest stained area, followed by those treated with LBA4404 and EHA105, displaying a similar trend to the GUS staining proportion (Figure 2h). Notably, the stained area ratio for GV3101 was statistically significantly higher than that of the other strains. Considering that GV3101 not only exhibited the highest staining proportion but also resulted in more intense and extensive staining, it was selected as the *Agrobacterium* strain for subsequent experiments.

#### 2.1.4. Plant Tissue

Histochemical GUS analysis was conducted to evaluate the transient transformation efficiency in different plant tissues, including leaves, cotyledons, and hypocotyls (Figure S3a–f). The results showed that leaves exhibited the highest staining proportion at 97.14%, followed by hypocotyls at 92.71% and cotyledons at 82.64%. Statistical analysis confirmed that the staining proportion in leaves was significantly higher compared to the other two tissues, suggesting that leaf is the most efficient tissue for transient transformation in *Cannabis sativa* ‘Cheungsam’ (Figure S3g). In addition, the blue-stained area was quantitatively analyzed using ImageJ software. The results revealed that the stained area ratio was also highest in leaves, followed by hypocotyls and cotyledons, showing a trend consistent with the GUS staining rate. However, unlike the staining proportion, the differences in stained area ratios among tissues were not statistically significant (Figure S3h).

#### 2.1.5. Application of Sonication and Vacuum Infiltration

**Figure 3:**
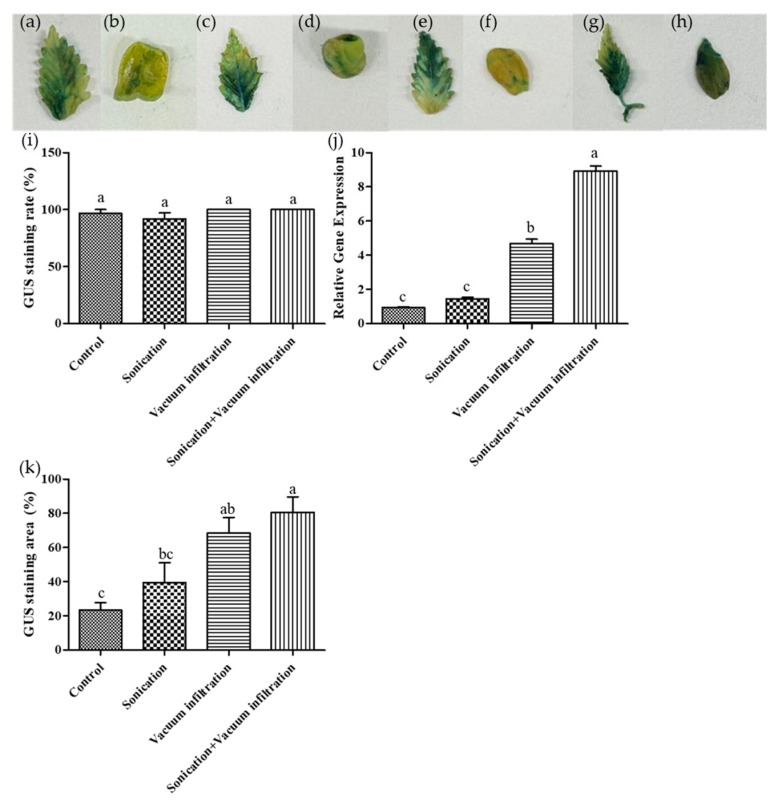
Effects of sonication and vacuum infiltration treatment on GUS expression in transformed plant tissues (leaf). (**a**–**h**) Representative images of histochemical GUS staining in different treatment groups. (**a**,**b**) untreated control; (**c**,**d**) sonication-only; (**e**,**f**) vacuum-infiltration only; (**g**,**h**) combination of sonication and vacuum infiltration. (**a**,**c**,**e**,**g**) leaf tissue, (**b**,**d**,**f**,**h**) cotyledon tissue. (**i**) Percentage of GUS-stained seedlings under various treatment conditions. (**j**) Relative GUS gene expression analyzed by qPCR. (**k**) Quantification of GUS-stained area under various treatments using ImageJ. Data represent the means of three replicates, and error bars indicate SEM. Means with the same letters are not significantly different (Tukey’s test, *p* < 0.05).

To evaluate the effects of sonication and vacuum infiltration treatments on transient transformation efficiency, four treatment groups were established using leaf tissues: a combination of sonication and vacuum infiltration, sonication alone, vacuum infiltration alone, and an untreated control (Figure 3a–h). The results showed that all treatment conditions exhibited staining proportions close to 100%. Specifically, the combination of sonication and vacuum infiltration, as well as the vacuum infiltration-only treatment, achieved the highest GUS staining proportions at 100%, followed by the untreated control at 96.67% and the sonication-only treatment at 91.67%. No statistically significant differences were observed among the treatments. However, treated samples exhibited a more intense GUS staining compared to the untreated control (Figure 3i).

In addition, the blue-stained area on the leaf surface was quantitatively measured using ImageJ software. The results showed that the combination of sonication and vacuum infiltration treatment resulted in the highest stained area ratio, followed by vacuum-infiltration only, sonication-only, and untreated control in descending order (Figure 3k).

Further qPCR analysis using leaf tissues confirmed the presence of *GUS* gene expression in all treatments. The highest GUS levels were observed in the combined sonication and vacuum infiltration treatment, which was statistically significant. This was followed by the vacuum-infiltration only treatment, the sonication-only treatment, and the untreated control in descending order. Results suggest that sonication induces micro-wounds in plant tissues, while vacuum infiltration treatment facilitates deeper infiltration of *Agrobacterium*, thereby enhancing transient transformation efficiency (Figure 3j).

### 2.2. Gene Expression Analysis of CBD Biosynthesis and TF Gene Following Transient Transformation

**Figure 4:**
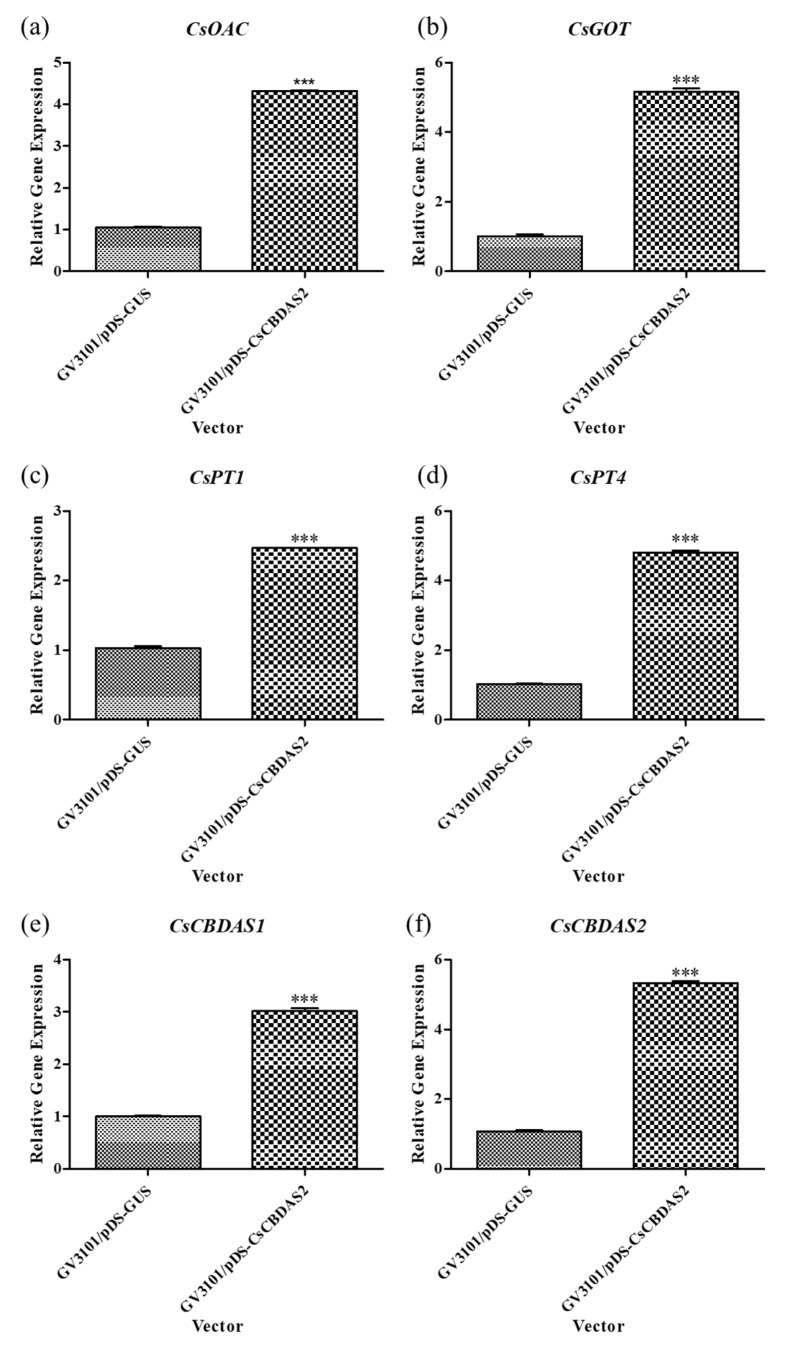
Comparison of the expression levels of CBD biosynthesis-related genes (**a**–**f**) in *Cannabis sativa* ‘Cheungsam’ plants overexpressing *CsCBDAS2* under dark conditions. Data represent the means of three replicates, and error bars indicate SEM. Statistical significance was determined using Student’s *t*-test, with *** *p* < 0.001.

**Figure 5:**
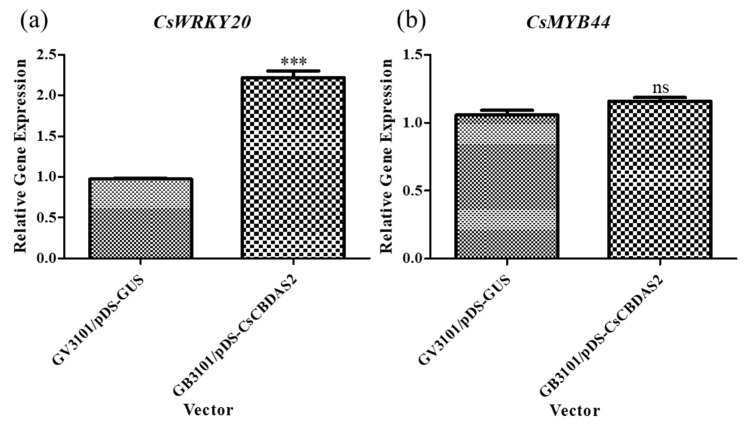
Comparison of the expression levels of transcription factor genes (**a**,**b**) in *Cannabis sativa* ‘Cheungsam’ plants overexpressing *CsCBDAS2* under dark conditions. Data represent the means of three replicates, and error bars indicate SEM. Statistical significance was determined using Student’s *t*-test, with ns (not significant), *** *p* < 0.001.

Based on previous experiments that established the optimal transient transformation method, we conducted further experiments using *Agrobacterium tumefaciens* GV3101 harboring the pDS-*CsCBDAS2* binary vector. The expression levels of genes related to CBD biosynthesis (*CsOAC*, *CsGOT*, *CsPT1*, *CsPT4*, *CsCBDAS1*, and *CsCBDAS2*), as well as the transcription factor (TF) *CsWRKY20*, were significantly upregulated in *Cannabis sativa* ‘Cheungsam’ treated with GV3101/pDS-*CsCBDAS2* compared to GV3101/pDS-*GUS* (Figure 4 and Figure 5a). In contrast, *CsMYB44* showed no statistically significant difference in expression levels between the GV3101/pDS-*CsCBDAS2* and GV3101/pDS-*GUS* treated groups (Figure 5b). These findings confirm that the transient transformation method established in this study is effective not only for introducing the GUS gene but also for successfully delivering target genes into the plant and inducing their expression.

## 3. Discussion

Transient transformation has been successfully performed in various *Cannabis sativa* cultivars and tissues in previous studies [26,27,28,29,30,31]. Most of these studies employed GUS staining for transformation confirmation, while a study by Kim et al. [30] provided the first insight into the subcellular localization of three key enzymes involved in CBD biosynthesis—*CsCBCAS*, *CsCBDAS*, and *CsTHCAS*—using recombinant vectors. In this study, we aimed to develop an optimized transient transformation protocol for industrial applications using the Korean hemp ‘Cheungsam’. To establish an optimal protocol, we systematically evaluated various experimental conditions.

First, we compared transformation efficiency using plants at different developmental stages (Figure S1). Specifically, 7-day-old and 21-day-old seedlings were tested, but no statistically significant differences were observed between the two groups. This finding aligns with previous reports, which utilized 10-day-old seedlings [27], 3–4-day-old seedlings [28], 1–3-week-old seedlings [29], and 1-week-old seedlings [31] as transformation materials. These studies collectively suggest that, despite variations in plant age across experiments, seedling age does not have a significant impact on transient transformation efficiency in *Cannabis sativa*.

Additionally, we evaluated the effect of light conditions on transformation efficiency by comparing the proportion of transformed plants grown under a 16 h/8 h (light/dark) condition with those grown in complete darkness (Figure S2). While no statistically significant differences in transient transformation proportion were detected between the two conditions, plants maintained in darkness exhibited more intense GUS staining.

Interestingly, most previous studies have utilized plants grown under a 16 h/8 h light cycle, and to our knowledge, no reports have explored the use of seedlings cultivated in complete darkness as transformation materials. This suggests that dark conditions might influence T-DNA uptake, expression efficiency, or histochemical staining intensity. Changes in light/dark photoperiod or light intensity may alter cell wall composition or epidermal cell thickness, potentially reducing *Agrobacterium* infiltration efficiency [32]. Further investigation into the role of photoperiod in *Agrobacterium*-mediated transformation of *Cannabis sativa* is warranted.

*Agrobacterium*-mediated transient transformation is a widely used approach for studying gene expression in plants. However, transformation efficiency varies significantly depending on the *Agrobacterium* strain used. In this study, we compared the transient transformation efficiency of multiple *Agrobacterium* strains, including GV3101, LBA4404, and EHA105. Among the tested strains, GV3101 exhibited the highest transformation efficiency, suggesting its suitability for transient gene expression in our experimental system (Figure 2). These findings indicate that transformation efficiency can vary substantially depending on the *Agrobacterium tumefaciens* strain employed.

Furthermore, strain selection may also depend on the plant variety. According to a previous study [27], LBA4404 and AGL1 exhibited higher transformation efficiency in the *Cannabis sativa* ‘Galaxy CBD’, whereas another study [31] reported GV3101 as the most efficient strain. These results highlight the importance of selecting an appropriate *Agrobacterium* strain for transient transformation, as both the bacterial strain and plant variety can influence transformation success.

We used GUS staining as an indicator of transformation success to evaluate the efficiency of transient transformation in various plant tissues, including leaves, hypocotyls, and cotyledons (Figure S3). The differences in transformation efficiency across plant tissues can be attributed to variations in cellular structure, physiological state, and responsiveness to *Agrobacterium* infiltration. Leaves, with their larger intercellular spaces and well-developed vascular networks, likely facilitate more efficient bacterial penetration and T-DNA delivery [33]. Additionally, the presence of actively dividing cells in young leaves may enhance transient gene expression, resulting in higher GUS activity. Our findings indicate that leaves are the optimal tissue for transient transformation in our system, providing higher transformation efficiency.

We applied sonication and vacuum infiltration treatments to *Cannabis* in order to enhance GUS staining efficiency and gene expression levels (Figure 3). All treatments exhibited high GUS staining proportion, with the most intense staining observed in the plants treated with both sonication and vacuum infiltration. Similarly, the highest GUS expression levels were found in the combination treatment group, followed by the vacuum-only treatment, sonication-only treatment, and the untreated control.

Sonication induces the formation of microscopic wounds in plant tissue, allowing *Agrobacterium* to penetrate plant cells more effectively [31,34]. This treatment creates additional entry points for *Agrobacterium*, thereby enhancing the agroinfiltration. Previous studies [29,35,36] have also reported that sonication treatment results in higher transformation efficiency compared to the untreated.

A previous study [37] has shown that vacuum infiltration improves transformation efficiency compared to non-vacuum methods. The vacuum treatment removes the air from the intercellular spaces in the submerged tissue through the stomata [38]. Once the vacuum is released and the internal pressure increases, the *Agrobacterium* suspension replaces the evacuated air and infiltrates the plant tissue. However, prolonged vacuum application can damage plant tissue, while insufficient vacuum time can result in inadequate air evacuation, reducing agroinfiltration efficiency [39].

Furthermore, studies have demonstrated that the combination of sonication and vacuum infiltration results in higher transformation efficiency compared to individual treatments or controls [31,40]. These results align with our findings, indicating that the combination of sonication and vacuum infiltration significantly enhances both GUS staining and GUS expression, thereby improving agroinfiltration efficiency in *Cannabis*.

The outcomes of this study highlight the potential of combining sonication and vacuum infiltration as an effective method to enhance transient gene expression in hemp and potentially other recalcitrant plants, offering insights into optimizing agroinfiltration protocols for efficacy improvement.

A recombinant vector containing *CsCBDAS2* was introduced into *Cannabis sativa* ‘Cheungsam’ using an optimized *Agrobacterium*-mediated transient transformation protocol (Figure 4 and Figure 5). qPCR analysis revealed that, in addition to *CsCBDAS2*, several CBD biosynthetic pathway-related genes (*CsOAC*, *CsGOT*, *CsPT1*, *CsPT4*, and *CsCBDAS1*) were significantly upregulated. Furthermore, among the two transcription factors examined, *CsWRKY20* expression was significantly increased, whereas *CsMYB44* showed no statistically significant change.

A previous study confirmed the transient expression of CBDAS and THCAS in *Cannabis sativa* using a CBDAS-recombinant vector [41]. Although CBDAS expression was significantly increased compared to the control, the study did not investigate changes in the expression levels of other biosynthetic genes or transcription factors.

In contrast, the present study demonstrates that *CsCBDAS2* overexpression not only increases CBDAS transcription but also induces the upregulation of upstream biosynthetic genes involved in precursor production. The upregulation of upstream biosynthetic genes upon *CsCBDAS2* overexpression implies that the pathway may be controlled by a tightly regulated transcriptional network, rather than functioning as a simple linear cascade. This finding suggests the presence of coordinated co-expression among genes in the CBD biosynthetic pathway. In a previous study [42], co-expression module analysis of genes involved in the cannabinoid biosynthetic pathway revealed that *CsaPT1* and *CsaPT4* were co-expressed with downstream pathway genes, including OAC and THCAS. Additionally, *CsaPT1* and *CsaPT4* exhibited the highest expression levels in trichome-specific tissues, where cannabinoid biosynthesis is most active. This regulatory coordination may help balance precursor supply and metabolic flux for efficient CBDA production.

Interestingly, while transcription factors typically act as upstream regulators of structural genes, our results suggest that the overexpression of a downstream biosynthetic gene (*CsCBDAS2*) may indirectly influence transcription factor activity, such as that of *CsWRKY20*. It is possible that metabolites derived from *CsCBDAS2* activity (e.g., CBGA or CBDA) function as signaling molecules that induce the expression of regulatory genes. A recent study demonstrated that CBDA and THCA accumulation under specific LED light treatments correlate with ROS stress-related signaling responses, suggesting that these metabolites may serve as indirect regulatory factors that modulate cannabinoid biosynthesis pathways [43]. This observation implies the existence of a positive feedback loop, where metabolic end-products influence upstream transcriptional regulation. WRKY20 transcription factors are well known to play roles in plant secondary metabolism, stress responses, and signaling cascades, and may therefore be integral components of the multi-layered regulatory network controlling cannabinoid biosynthesis [44].

Taken together, the coordinated upregulation of both CBD biosynthetic enzymes (*CsOAC*, *CsGOT*, *CsPT1*, *CsPT4*, *CsCBDAS1*, and *CsCBDAS2*) and the transcription factor *CsWRKY20* upon *CsCBDAS2* overexpression highlights the need for further molecular and functional studies to elucidate the precise regulatory mechanisms governing cannabinoid biosynthesis in *Cannabis sativa*. Furthermore, the optimized transient transformation conditions established in this study provide a robust platform for investigating these complex regulatory networks.

## 4. Materials and Methods

### 4.1. Seed Sterilization and Germination

*Cannabis sativa* ‘Cheungsam’ seeds were provided by the cultivation farmland of the Korea Hemp Industry Association, an incorporated association located in Imha-myeon, Andong-Si, Gyeongsangbuk-do, Republic of Korea.

Seed germination of the plants was confirmed following the protocol described by Baek et al. [45]. Briefly, seeds of *Cannabis sativa* ‘Cheungsam’ were surface sterilized with 10% sodium hypochlorite (Daejung, Siheung, Republic of Korea) solution for 10 min, before being rinsed with sterile distilled water 5 to 8 times. The seeds were then subjected to sonication (40 kHz) for 10 min. After sonication, the seeds were primed with 1% hydrogen peroxide (H_2_O_2_, Daejung, Republic of Korea) solution and cultivated in darkness (25 ± 2 °C) for 3 days. After 7 days or 21 days of culture, we used these explants. The seeds were germinated on MS supplemented with Gamborg B5 vitamins (Duchefa, The Netherlands), 3% sucrose (MB cell, Seoul, Republic of Korea), and 0.8% plant agar (Duchefa, The Netherlands). The pH was adjusted to 5.8 with 1N NaOH or HCl and autoclaved at 121 °C for 15 min. All cultures were maintained at 25 ± 2 °C under 16 h/8 h (light/dark) photoperiod at the light intensity of 120.25 µmol m^−2^s^−1^ LED daylight or darkness.

### 4.2. Vector Construction and Transformation by Agrovacterium

The *CsCBDAS2* and *GUS* genes were cloned into a binary vector using the Gateway cloning system (Thermo Fisher Scientific, Waltham, MA, USA). Initially, the coding sequences of these genes were amplified by polymerase chain reaction (PCR) using gene-specific primers containing attB recombination sites. The amplified fragments were purified and recombined into the pDONR221 entry vector via a BP recombination reaction using BP Clonase II (Invitrogen, Waltham, MA, USA), following the manufacturer’s protocol. The entry clones were verified by colony PCR and Sanger sequencing services provided by Macrogen Inc. (Seoul, Republic of Korea).

**Figure 6:**
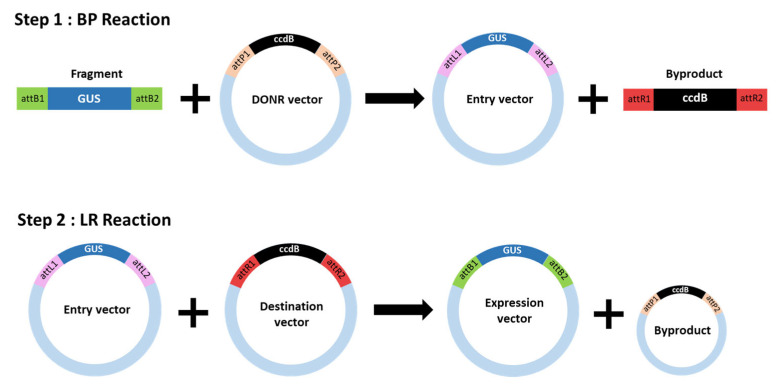
Schematic representation of the Gateway cloning system.

**Figure 7:**
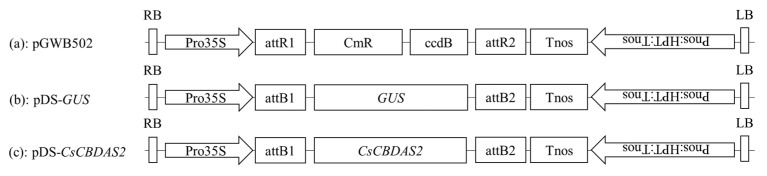
Schematic of the T-DNA structure of the binary vectors used for transient gene expression. (**a**) pGWB502; (**b**) *GUS* gene; and (**c**) *CsCBDAS2* gene.

Subsequently, the confirmed entry clones were recombined into the destination binary vector pGWB502 using LR Clonase II (Invitrogen, USA). The resulting expression constructs contained the target genes under the control of the CaMV 35S promoter and were verified through restriction enzyme digestion and sequencing (Figure 6 and Figure 7).

The final constructs of *CsCBDAS2* and *GUS* were introduced into *Agrobacterium tumefaciens* strain GV3101 using the freeze–thaw method as described by Jyothishwaran et al. [46]. Additionally, the GUS construct was also transformed into LBA4404 and EHA105 strains using the same method.

### 4.3. Agrobacterium Cell Culture and Preparation

The transformed *Agrobacterium* strains were grown on LB agar (Duchefa Biochemie, Noord-Holland, The Netherlands) plates containing various antibiotics at 28 °C for 2 days (Table 1).

A single colony was selected and inoculated into 5 mL of liquid LB (Duchefa Biochemie, The Netherlands) medium supplemented with the same antibiotics. The culture was incubated at 28 °C with shaking at 200 rpm overnight until the OD_600_ = 0.5–1.0. This overnight culture was diluted (1:50) into fresh LB medium containing 20 µM 3′,5′-Dimethoxy-4′-hydroxyacetophenone (acetosyringone, Sigma Aldrich, St. Louis, MO, USA) and 10 mM MES-KOH (Sigma Aldrich, USA), and incubated under the same conditions until OD_600_ = 0.8–1.0.

Cells were harvested by centrifugation at 2800× *g* for 10 min at room temperature and resuspended in an infiltration buffer consisting of 10 mM MgCl_2_ (Sigma Aldrich, USA), 10 mM MES-KOH (pH 5.6), and 0.01% Silwet L-77 (PhytoTech Labs, Lenexa, KS, USA). The final OD_600_ of the *Agrobacterium* suspension was adjusted to 0.5 before infiltration. The suspension was incubated at room temperature for 3 h, and 100 µM acetosyringone was added to the suspension immediately before infiltration to enhance penetration.

### 4.4. Co-Cultivation

**Figure 8:**
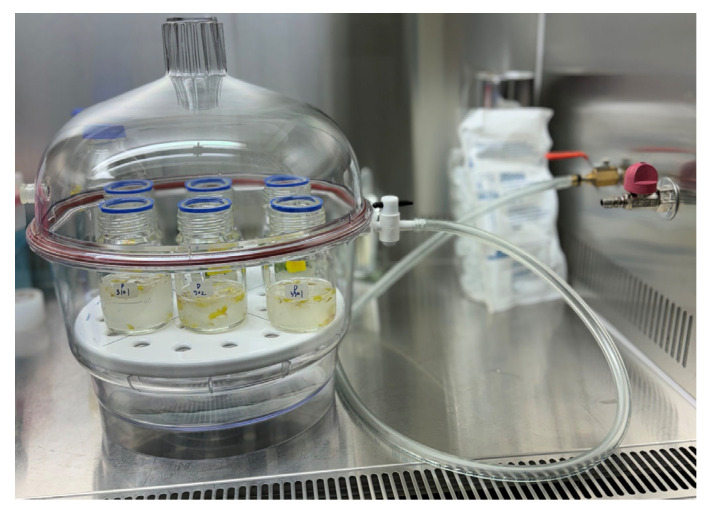
Vacuum applied to seedlings submerged in *Agrobacterium* cell suspension.

**Figure 9:**
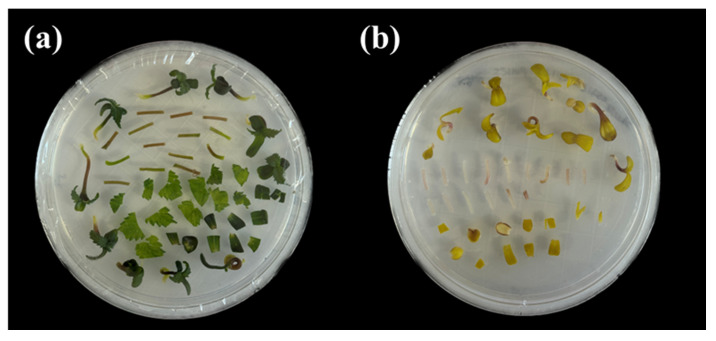
Explants that were transferred to MS medium after co-cultivation. (**a**) 16 h/8 h (light/dark) condition; (**b**) dark condition.

Plant tissues were divided into leaves, cotyledons, and hypocotyls, and these were precisely cut using a scalpel. The tissue segments were then placed in 40 mL of infiltration buffer containing *Agrobacterium* and subjected to sonication for 30 s. After sonication, vacuum infiltration was performed using a desiccator (Sigma-Aldrich, USA) and a pump for 20 min (Figure 8). Following the vacuum treatment, the samples were blot-dried using sterile filter paper to remove extra liquid. Subsequently, the explants were transferred to the MS medium (Figure 9). The initial incubation was conducted at 22 °C under dark conditions for 3 days, followed by further incubation at 24 °C with a 16 h/8 h (light/dark) photoperiod for an additional 2 days. After 2 days, the explants were washed 3 times with sterile distilled water containing 300 mg/L Timentin (Gold bio, St. Louis, MO, USA) for 1 min, then blotted dry using sterilized filter paper.

### 4.5. Histochemical Detection of GUS Activity

To analyze GUS expression, tissue samples were first surface washed and immersed in pre-chilled 90% acetone (Junsei, Tokyo, Japan). The samples were then subjected to vacuum infiltration at room temperature for 10 min, followed by fixation at room temperature for 30 min. After fixation, the acetone was removed, and wash buffer containing 0.5 M EDTA (Biosesang, Yongin, Republic of Korea), 100 mM Phosphate buffer (Sigma-Aldrich, USA), 10% Triton X-100 (Sigma-Aldrich, USA), 100 mM potassium ferrocyanide (Duksan, Seoul, Republic of Korea), 100 mM potassium ferricyanide (Duksan, Republic of Korea), and 2 mM methanol (Daejung, Republic of Korea) was added. The samples were placed on ice and subjected to vacuum infiltration for 10 min. Following the wash step, the buffer was removed, and staining buffer containing 0.5 M EDTA, 100 mM Phosphate buffer, 10% Triton X-100, 100 mM potassium ferrocyanide, 100 mM potassium ferricyanide, 2 mM methanol, and 100 mM X-Gluc (Bio Plus, Daejeon, Republic of Korea) was added. The samples were kept on ice and were vacuum infiltrated for 20 min. After releasing the vacuum, an additional vacuum infiltration step was performed for another 20 min. The samples were then incubated at 37 °C for 24 h to allow for GUS staining.

After removing the staining buffer, the samples were sequentially treated with 20%, 35%, and 50% ethanol at room temperature for 30 min each. Following the ethanol series, the samples were incubated in FAA solution containing 50% ethanol, 37% formaldehyde (Sigma-Aldrich, USA), and 5% acetic acid (Daejung, Republic of Korea) at room temperature for 30 min. The FAA solution was discarded, and the samples were transferred to 70% ethanol. The treated samples were subsequently examined under both the optical camera and a light microscope (Olympus, Tokyo, Japan).

### 4.6. Quantification of GUS-Stained Area Using ImageJ

Histochemically GUS-stained seedlings were photographed under identical lighting conditions using a digital camera. The images were then analyzed using ImageJ software (NIH, Bethesda, MD, USA) to quantify the blue-stained area. To eliminate background interference, the images were first converted to 8-bit grayscale and then subjected to threshold adjustment to isolate the stained regions. The region of interest (ROI) was manually selected to define the total leaf area, and the percentage of GUS-stained area was calculated by dividing the stained pixel count by the total pixel count within the ROI.

### 4.7. Expression Analysis of Genes Related to GUS, CBD Biosynthesis, and TF Genes

Total RNA was extracted from the tissue of plants using the Trizol^®®^ Reagent (Invitrogen, USA), following the manufacturer’s instructions. An oligo (dT) 20-mer primer was used for reverse transcription of the RNA into complementary DNA (cDNA) using an AccuPower^®^ RT PreMix (Bioneer, Daejeon, Republic of Korea), following the manufacturer’s instructions. Next, the expression levels of *GUS*, CBD biosynthesis genes [olivetolic acid cyclase (*OAC*), geranylpyrophosphate:olivatolate geranyltransferase (*GOT*), prenyltransferase 1 (*PT1*), prenyltransferase 4 (*PT4*), cannabidiolic acid synthase 1 (*CBDAS1*), cannabidiolic acid synthase 2 (*CBDAS2*)], and transcription factors (*WRKY20* and *MYB44*) were detected using the LightCycler^®^ 96 Real-Time PCR system (Roche, Basel, Switzerland). The PCR conditions and primers used for Quantitative real-time PCR (qPCR) are shown in (Table 2). Gene expression levels were measured using the 2^−ΔΔ^Ct method, based on three independent biological replications [47]. The Protein Phosphatase 2A (*PP2Aq*) gene was used as the housekeeping gene.

### 4.8. Statistical Analysis

All statistical analyses were performed using GraphPad Prism version 5.03 (GraphPad Software, San Diego, CA, USA). For comparisons involving more than two groups, a one-way analysis of variance (ANOVA) was conducted, followed by Tukey’s multiple comparison test for post-hoc analysis. For comparisons between two groups, an independent two-tailed Student’s *t*-test was performed. Statistical significance was set at *p* < 0.05. All experiments were conducted in three replicates. Data are presented as mean ± standard error of the mean (SEM) unless otherwise stated.

## 5. Conclusions

This study presents a reliable and efficient transient transformation protocol for *Cannabis sativa* ‘Cheungsam’, optimized through a systematic evaluation of multiple influencing factors. We demonstrated that early-stage seedlings, dark cultivation conditions, the GV3101 *Agrobacterium* strain, leaf tissue, and combined sonication and vacuum treatments contribute to high transformation efficiency.

Functional analysis using a *CsCBDAS2*-expressing vector confirmed successful gene delivery and expression, and revealed that *CsCBDAS2* overexpression induces the upregulation of upstream biosynthetic genes and the transcription factor *CsWRKY20*. These findings suggest that the CBD biosynthetic pathway may be regulated by a complex gene co-expression network and potentially a positive feedback mechanism. Our work not only advances the toolkit for functional genomics in hemp but also lays a foundation for future metabolic engineering and synthetic biology applications in cannabinoid research.

## Figures and Tables

**Table 1:** Antibiotic sensitivity of different vectors.

Vector	Antibiotics			
	Rifampicin	Spectinomycin	Streptomycin	Gentamicin
	25 µg/mL	50 µg/mL	50 µg/mL	25 µg/mL
GV3101/pGWB502	Resistant	Resistant	Sensitive	Resistant
LBA4404/pGWB502	Resistant	Resistant	Resistant	Sensitive
EHA105/pGWB502	Resistant	Resistant	Resistant	Sensitive

**Table 2:** Primer sequences and conditions used for quantitative real-time PCR analysis of gene expression.

Gene	Forward Primer (5′ to 3′)	Reverse Primer (5′ to 3′)
*GUS*	TCAGCAAGCGCACTTACA	ATAACGGTTCAGGCACAGC
*CsOAC*	CACAGAAGCCCAAAAGGAAG	CAACATGGGCAGGATGAATA
*CsGOT*	GCGCCAAGCAGACAATTCTT	ATGGCCTCCCCTTGTAGTGA
*CsPT1*	ATCGACACCCCAAAACACCA	TCCCAAACAATCCACAAGCG
*CsPT4*	ACGCCAAATATGGGGTATCA	TTGCTAGAGCAAGCTCACGA
*CsCBDAS1*	GCCAAACTGCATGGGTTGAA	CCTCCACCACCACGTATAGC
*CsCBDAS2*	AGGTGGACACTTTGGTGGAG	TGATTCCGAAGCTTTCTGCT
*CsWRKY20*	GTCCCTGCTGCGAGAAATAG	TCAGATTGCGCAGTAGGATG
*CsMYB44*	TATTGTTGCAGCGAGAACCA	ATCTTGCCAAGTCCCATACG
*CsPP2Aq*	AGCAACGTTCAGCCCGTTAA	GACACTTCCCTCCAATTCGAAA

PCR condition: 95 °C (180 s) followed by 45 cycles of [95 °C (10 s), 56 °C (10 s), 72 °C (20 s)], 95 °C (10 s), 65 °C (60 s), 97 °C (1 s), 37 °C (30 s).

## Data Availability

All data are contained in this article.

## Supplementary Materials

The following supporting information can be downloaded at https://www.mdpi.com/article/10.3390/plants14101460/s1, Figure S1: Effect of developmental stages on GUS expression in transformed *Cannabis sativa* ‘Cheungsam’ tissues; Figure S2: Effect of light condition on GUS expression in transgenic *Cannabis sativa* ‘Cheungsam’ tissues; Figure S3: GUS expression in different *Cannabis sativa* ‘Cheungsam’ tissues.

## Abbreviations

The following abbreviations are used in this manuscript:

CBD	Cannabidiol
TF	Transcription factor
THC	∆^9^-tetrahydrocannabinol
CBDA	Cannabidiolic acid
CBGA	Cannabigerolic acid
THCA	Tetrahydrocannabinolic acid
CBDAS	CBDA synthase
THCAS	THCA synthase
OA	Olivetolic acid
GPP	Geranylpyrophosphate
FAD	Flavin adenine dinucleotide
T-DNA	Transfer DNA
VIGS	Virus-induced gene silencing
TMV	Tobacco mosaic virus
PCR	Polymerase chain reaction
cDNA	Complementary DNA
OAC	Olivetolic acid cyclase
GOT	Geranylpyrophosphate:olivatolate geranyltransferase
PT1	Prenyltransferase 1
PT4	Prenyltransferase 4
CBDAS1	Cannabidiolic acid synthase 1
CBDAS2	Cannabidiolic acid synthase 2
qPCR	Quantitative real-time
PP2Aq	Protein Phosphatase 2A
SEM	Standard error of the mean
Acetosyringone	3′,5′-Dimethoxy-4′-hydroxyacetophenone
