# Supplementary material for: CsCBDAS2-Driven Enhancement of Cannabinoid Biosynthetic Genes Using a High-Efficiency Transient Transformation System in Cannabis sativa ‘Cheungsam’
Source: Plants (Basel). 2025 May 14;14(10):1460. doi: 10.3390/plants14101460 (PMC12114937; doi:10.3390/plants14101460)
Supplement: Supplementary file 1 [file plants-14-01460-s001.zip › plants-3587061-supplementary.pdf]

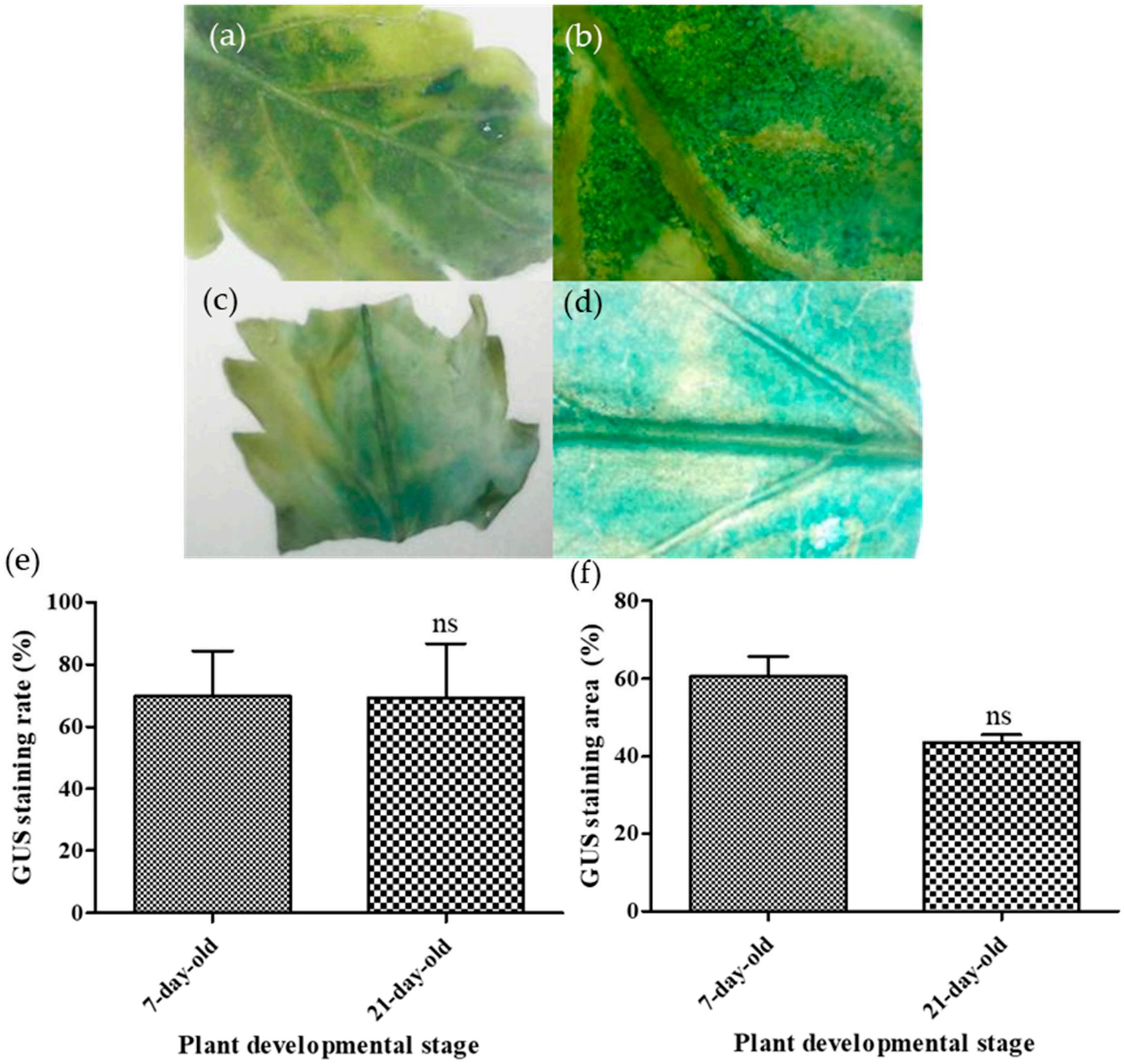

Figure S1. Effect of developmental stages on GUS expression in transformed *Cannabis sativa* 'Cheungsam' tissues. (a) – (d): Representative images of histochemical GUS staining in seedlings at different developmental stages; (a), (b): 7-day-old seedlings; (c), (d): 21-day-old seedlings; (e): Percentage of GUS-stained seedling at 7 and 21 days after transformation. (f): Quantification of GUS-stained area in tissues using ImageJ. Data represent the means of three replicates, and error bars indicated SEM. Statistical significance was determined using Student's t-test, with ns (not significant).

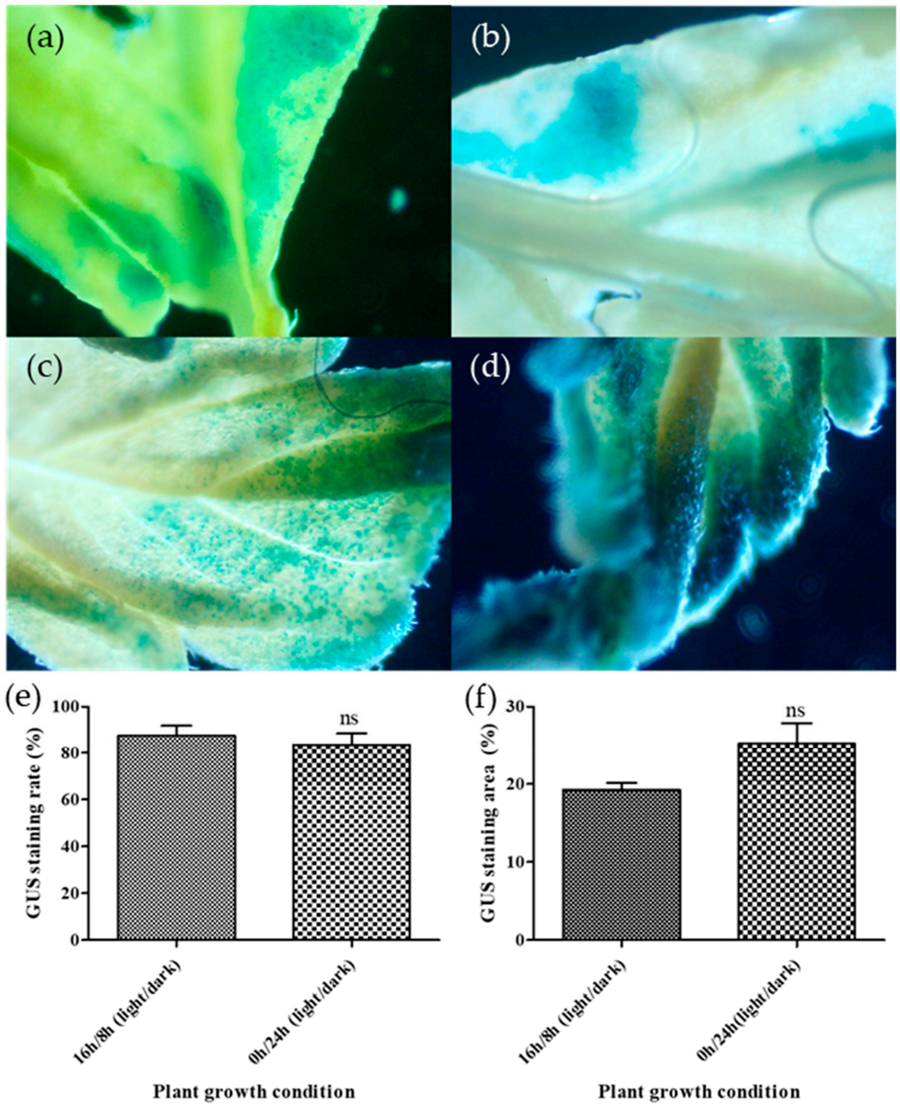

Figure S2. Effect of light condition on GUS expression in transgenic *Cannabis sativa* 'Cheungsam' tissues. (a) - (b): Representative images of histochemical GUS staining in plants grown under 16/h/8h (light/dark). (c) – (d): Plant grown under dark condition. (e): Percentage of GUS-stained seedlings under different light conditions after transformation. (f): Quantification of GUS-stained area in tissues using ImageJ. Data represent the means of three replicates and error bars indicate SEM. Statistical significance was determined using Student's t-test, with ns (not significant).

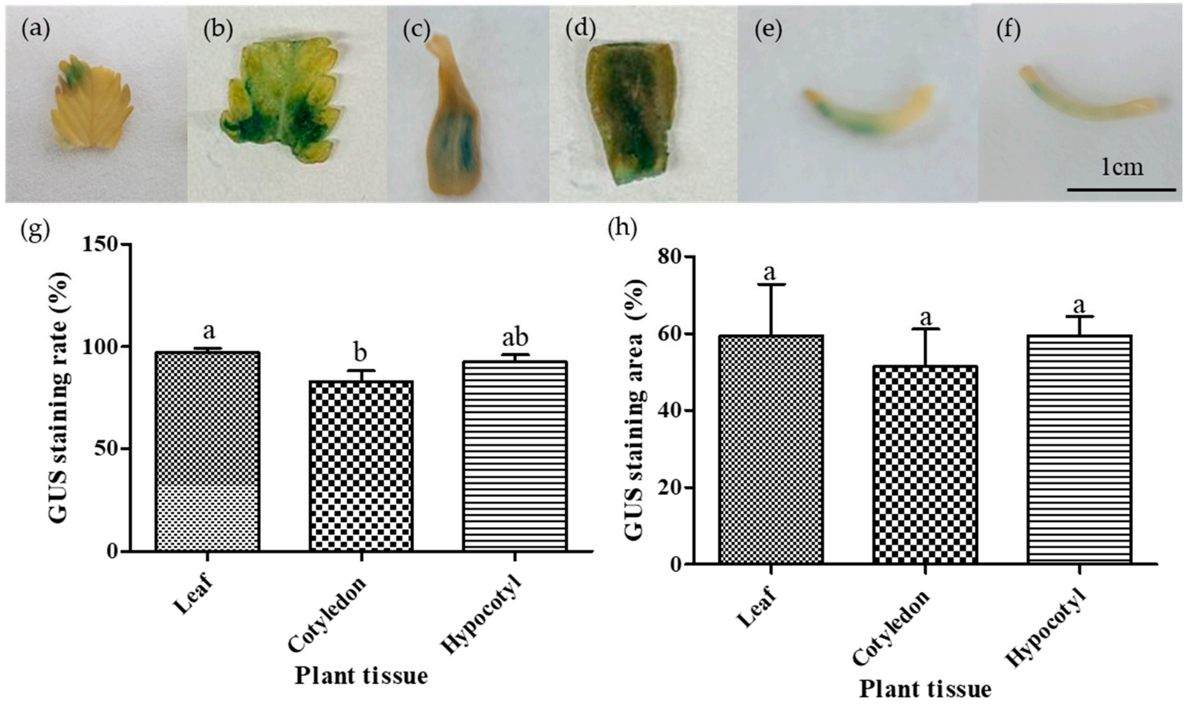

Figure S3. GUS expression in different *Cannabis sativa* 'Cheungsam' tissues. (a) – (f): Representative images of histochemical GUS staining. (a), (b): leaf, (c), (d): cotyledon, (e), (f): hypocotyl. Scale bars = 1cm. (g): Percentage of GUS-stained different plant tissues after transformation. (h): Quantification of GUS-stained area in tissues using ImageJ. Data represent the means of three replicates and error bars indicate SEM. Means with the same letters are not significantly different (Tukey's test, $p < 0.05$).
